# Expression and Function of K(ATP) Channels in Normal and Osteoarthritic Human Chondrocytes: Possible Role in Glucose Sensing

**DOI:** 10.1002/jcb.24532

**Published:** 2013-03-13

**Authors:** Ana T Rufino, Susana C Rosa, Fernando Judas, Ali Mobasheri, M Celeste Lopes, Alexandrina F Mendes

**Affiliations:** 1 Center for Neuroscience and Cell Biology, University of Coimbra3004-517 Coimbra, Portugal; 2 Faculty of Pharmacy, University of Coimbra3000-548 Coimbra, Portugal; 3 Orthopaedics Department, University Hospital of Coimbra3000-075 Coimbra, Portugal; 4 Faculty of Medicine, University of Coimbra3000-548 Coimbra, Portugal; 5 Arthritis Research UK Centres for Pain, Musculoskeletal Ageing Research and Sport, Exercise and Osteoarthritis, University of Nottingham, Sutton Bonington CampusLeicestershire LE12 5RD, United Kingdom; 6 Center of Excellence in Genomic Medicine Research, King Fahad Medical Research Center, King Abdulaziz UniversityJeddah 21589, Kingdom of Saudi Arabia

**Keywords:** ARTICULAR CARTILAGE, OSTEOARTHRITIS, K(ATP) CHANNEL, GLUCOSE TRANSPORTER, HUMAN CHONDROCYTE, HYPERGLYCEMIA

## Abstract

ATP-sensitive potassium [K(ATP)] channels sense intracellular ATP/ADP levels, being essential components of a glucose-sensing apparatus in various cells that couples glucose metabolism, intracellular ATP/ADP levels and membrane potential. These channels are present in human chondrocytes, but their subunit composition and functions are unknown. This study aimed at elucidating the subunit composition of K(ATP) channels expressed in human chondrocytes and determining whether they play a role in regulating the abundance of major glucose transporters, GLUT-1 and GLUT-3, and glucose transport capacity. The results obtained show that human chondrocytes express the pore forming subunits, Kir6.1 and Kir6.2, at the mRNA and protein levels and the regulatory sulfonylurea receptor (SUR) subunits, SUR2A and SUR2B, but not SUR1. The expression of these subunits was no affected by culture under hyperglycemia-like conditions. Functional impairment of the channel activity, using a SUR blocker (glibenclamide 10 or 20 nM), reduced the protein levels of GLUT-1 and GLUT-3 by approximately 30% in normal chondrocytes, while in cells from cartilage with increasing osteoarthritic (OA) grade no changes were observed. Glucose transport capacity, however, was not affected in normal or OA chondrocytes. These results show that K(ATP) channel activity regulates the abundance of GLUT-1 and GLUT-3, although other mechanisms are involved in regulating the overall glucose transport capacity of human chondrocytes. Therefore, K(ATP) channels are potential components of a broad glucose sensing apparatus that modulates glucose transporters and allows human chondrocytes to adjust to varying extracellular glucose concentrations. This function of K(ATP) channels seems to be impaired in OA chondrocytes. J. Cell. Biochem. 114: 1879–1889, 2013. © 2013 Wiley Periodicals, Inc.

Glucose serves an important anabolic function in chondrocytes, being required for the synthesis of matrix components. Due to the avascular nature of cartilage, chondrocytes are physiologically exposed to low oxygen tensions [Zhou et al., 2004; Grimshaw and Mason, 2000; Kay et al., 2011], using glycolysis as the main energy source [Rajpurohit et al., 1996], even when exposed to normoxia [Schneider et al., 2007]. The supply of glucose to chondrocytes depends on diffusion from the synovial fluid [Maroudas, 1970] which is facilitated by joint motion and fluid flow in the extracellular matrix (ECM). The synovial fluid glucose concentration is identical to that in the plasma both in normal conditions and in non-inflammatory and inflammatory types of arthritis, except in septic arthritis where it is usually significantly reduced [Brannan and Jerrard, 2006]. Several members of the facilitative glucose transporter family—the GLUT/SLC2A transporters—are expressed in chondrocytes and optimize its uptake [Mobasheri et al., 2008]. GLUT-1 is especially important as both anabolic and catabolic stimuli increase its expression, while others, like GLUT-3, which is also highly expressed constitutively, does not seem to be regulated by those stimuli [Richardson et al., 2003; Shikhman et al., 2004; Phillips et al., 2005; Mobasheri et al., 2008].

On the other hand, extracellular glucose concentrations both below and above physiologic levels, have been predicted and shown to alter the pattern of gene expression [Mobasheri et al., 2002; Rosa et al., 2011], as well as the availability of GLUT-1 protein [Rosa et al., 2009] in chondrocytes. Indeed, glucose deprivation increases the expression of GLUT-1, whereas a supra-physiologic glucose concentration has the opposite effect, decreasing GLUT-1 content and glucose uptake in young/healthy chondrocytes, but has no effect on those isolated from osteoarthritic (OA) cartilage [Rosa et al., 2009]. Therefore, normal chondrocytes seem to be able to adjust to the extracellular glucose concentration by modulating the abundance of GLUT-1 and the total glucose transport capacity. Nonetheless, this ability seems to be compromised in OA chondrocytes in vitro, leading to intracellular glucose accumulation, increased production of reactive oxygen species [Rosa et al., 2009] and expression of matrix degrading enzymes [Rosa et al., 2011] when these cells are exposed to supra-physiologic glucose concentrations, as those occurring in diabetes mellitus, metabolic syndrome and other conditions characterized by impaired glucose metabolism. These conditions are increasingly recognized as independent risk factors for OA development and progression [Hart et al., 1995; Sturmer et al., 2001; Del Rosso et al., 2006; Katz et al., 2010; Velasquez and Katz, 2010; Berenbaum, 2011; Schett et al., 2013]. OA is the major musculoskeletal disorder—or most likely, a set of disorders with distinct etiological and clinical characteristics [Berenbaum, 2011; Zhuo et al., 2012]—and a main cause of physical disability [Martel-Pelletier et al., 2008; Goldring and Marcu, 2009]. Understanding how human chondrocytes sense extracellular glucose and intracellular ATP concentrations and adjust their substrate uptake to match the cell's needs and whether such mechanisms remain functional in OA chondrocytes is, thus, critical to elucidate the mechanisms by which conditions associated with impaired glucose metabolism contribute to the development and/or progression of OA.

ATP-sensitive potassium [K(ATP)] channels are weak inwardly rectifying potassium channels that act as metabolic sensors in a number of cell types, coupling metabolic activity to the membrane potential and to secretory activity, primarily by sensing intracellular ATP levels [Miki et al., 2002; Minami et al., 2004; Burke et al., 2008; McTaggart et al., 2010]. In pancreatic β-cells [Burke et al., 2008] and in subpopulations of mediobasal hypothalamic neurons [Acosta-Martinez and Levine, 2007], for instance, K(ATP) channels have been identified as an essential component of a glucose-sensing mechanism, wherein uptake and metabolism of glucose leads to increased intracellular ATP/ADP levels, closure of the channels, and increase in insulin secretion and neuronal excitability, respectively.

Structurally, K(ATP) channels are heteromultimers of four ATP-binding cassette (ABC) proteins that form the sulfonylurea receptor (SUR), surrounding four pore forming potassium channel subunits (Kir6) [Babenko et al., 1998]. Kir6 and SUR have two isoforms each, Kir6.1 and Kir6.2 and SUR1 and SUR2, respectively. SUR2 has two major splice variants, SUR2A and SUR2B [Burke et al., 2008; Hibino et al., 2010]. ATP binds to the SUR subunits inducing a conformational change of the Kir6 subunits that results in channel closure, cessation of K^+^ efflux and cell membrane depolarization. In contrast, decreased intracellular ATP availability allows ADP binding and channel opening with the consequent K^+^ efflux and hyperpolarization [Babenko et al., 1998; Shi et al., 2005].

The subunit composition of K(ATP) channels determines their sensitivity to intracellular ATP and to pharmacological modulators, eliciting the functional diversity of these channels [Babenko et al., 1998; Inagaki and Seino, 1998; Acosta-Martinez and Levine, 2007]. Kir6.1 and Kir6.2 have similar sensitivities to nucleotides (ATP and ADP), so the differences found are believed to be due to the SUR subtype existing in each channel or to the combination of different SUR subunits in the same channel [Shi et al., 2005; Chan et al., 2008;]. K(ATP) channels involved in coupling glucose sensing with insulin secretion in pancreatic β-cells are composed of Kir6.2 and SUR1 [Nielsen et al., 2007], whereas channels containing Kir6.2 and SUR2A, found in cardiomyocytes, and channels composed of Kir6.2 or Kir6.1 and SUR2B, occurring in vascular cells, regulate the duration of the action potential and vasodilation [Aguilar-Bryan and Bryan, 1999; Gribble and Ashcroft, 2000; Burke et al., 2008; Chan et al., 2008; Hibino et al., 2010].

Information regarding the subunit composition and function of K(ATP) channels expressed in non-excitable cells is much scarcer. Functional K(ATP) channels have been demonstrated in equine chondrocytes and Kir6.1 was detected in normal and OA human cartilage [Mobasheri et al., 2007], but their subunit composition and physiological roles in cartilage are not yet understood, even though these channels are highly likely to be important for regulation of cartilage metabolism and sensing ATP levels within the cell [Barrett-Jolley et al., 2010; Mobasheri et al., 2012]. We hypothesize that K(ATP) channels, by responding to ATP levels, may indirectly act as components of the glucose sensing apparatus in chondrocytes, generating signals that allow the cell to adjust to varying extracellular glucose concentrations by regulating the availability of its facilitative glucose transporters and hence net glucose transport capacity. Therefore, this work was aimed at (i) elucidating the subunit composition of K(ATP) channels expressed in human chondrocytes and (ii) determining whether K(ATP) channels play a role in regulating the abundance of two major glucose transporters in chondrocytes, GLUT-1 and GLUT-3, and the glucose transport capacity of human chondrocytes exposed to hyperglycemia-like conditions. Furthermore, these processes were evaluated in chondrocytes isolated from cartilage samples with different degradation grades to determine whether they are impaired in OA.

## MATERIALS AND METHODS

### Cartilage Samples

Human knee cartilage was collected, within 12 h of death, from the distal femoral condyles of multi-organ donors (24–70 years old, mean = 49.9, n = 20) or with informed consent from patients (60–76 years old, mean = 67.7, n = 10) undergoing total knee replacement surgery at the Orthopaedic Department of the University Hospital of Coimbra (HUC). The Ethics Committee of HUC approved all procedures. The cartilage samples were divided in three groups according to the degree of macroscopic damage, using the classification of Outerbridge (1961). Groups 0–1 (n = 11, 24–70 years old, mean = 41.5), includes cartilage samples with no macroscopic signs of degradation (score 0) or presenting only a softened surface (score 1); Groups 2–3 (n = 9, 47–70 years old, mean = 60.1) includes fibrillated or fissured cartilage samples without evident signs of surface erosion (scores 2 and 3); and Group 4 (n = 10, 60–76 years old, mean = 67.7) comprises severely damaged cartilage samples, with areas of extensive full thickness erosion that exposed the subchondral bone, corresponding to advanced OA.

### Chondrocyte Isolation and Culture

Chondrocytes were isolated by enzymatic digestion from non-pooled cartilage samples, as described previously [Rosa et al., 2008]. To avoid dedifferentiation, confluent non-proliferating monolayer cultures were established from each cartilage sample, allowed to recover in Ham F-12 medium containing 5% fetal bovine serum for 24 h, serum-starved for at least 6 h and maintained thereafter in serum-free culture medium. Serum-starved chondrocyte cultures were treated with glibenclamide (Tocris Biosciences, UK) or high glucose as indicated in Results Section and in legends of figures and tables.

### Assessment of Cell Viability

The 3-(4,5-dimethylthiazol-2-yl)-2,5-diphenyltetrazolium (MTT, Sigma Chemical Co., St. Louis, MO) reduction assay [Mosmann, 1983] was used to evaluate cell viability in order to assess possible cytotoxic effects of culturing chondrocyte under hyperglycemia-like conditions. Chondrocyte cultures treated or not with high glucose (30 mM) for 18 h were incubated, for 1 h, at 37°C, with a 0.5 mg/ml MTT solution in Ham F-12 medium. The dark blue crystals of formazan produced were dissolved in acidified isopropanol, and formazan quantification was performed by measuring the absorbance of the corresponding solution, using an automatic plate reader (SLT, Austria) set at a test wavelength of 570 nm and a reference wavelength of 620 nm.

### Western Blotting

Whole cell extracts were prepared as previously described [Rosa et al., 2009]. The proteins in the extracts (25 µg) and molecular weight markers (All blue, Precision Plus molecular weight markers, Bio-Rad Laboratories Inc., Hercules, CA) were separated by SDS–PAGE and transferred to PVDF membranes by electroblotting. Membranes were probed with Rabbit polyclonal antibodies against human GLUT-1 and GLUT-3 (Millipore, Tremecula, CA), mouse monoclonal anti-human SUR1 (Abcam, Cambridge, UK), goat polyclonal anti-human SUR2A, SUR2B, and Kir6.1 and rabbit polyclonal anti-human Kir6.2 (Santa Cruz Biotechnology, Inc., Santa Cruz, CA) antibodies and then with an alkaline phosphatase-conjugated secondary antibody. Immune complexes were detected using the Enhanced ChemiFluorescence reagent (GE Healthcare, UK) in a Typhoon FLA 9000 scanner (GE Healthcare). A mouse anti-human β-tubulin monoclonal antibody (Sigma–Aldrich, Co., St. Louis, MO) was used to detect β-tubulin as a loading control. The intensity of the bands was analyzed using ImageQuant™ TL (version 7.0, GE Healthcare). The results were normalized by calculating the ratio between the intensities of the bands corresponding to the protein of interest and β-tubulin.

### Immunohistochemistry

Five millimeters of diameter full depth cartilage cores were collected from the distal femoral condyles of multi-organ donors, immersed in OCT embedding compound (TAAB Laboratories, UK) and immediately frozen at −80°C. Cryostat sections, 10 µm thick, were fixed in acetone at −20°C for 10 min and incubated with the primary antibodies against Kir6.1, Kir6.2, SUR1, SUR2A, and SUR2B mentioned in the Western blot section. Secondary antibodies used were Alexa fluor 488-conjugated goat anti-rabbit, Alexa fluor 488-conjugated goat anti-mouse (Molecular Probes, Eugene, OR), and FITC-conjugated rabbit anti-goat (Serotech Laboratories, Toronto, Canada). Counterstaining was performed with DAPI (Sigma–Aldrich) to allow nuclear visualization. Specificity was assessed by omitting the first antibody (negative control). Images of cartilage sections were acquired in a confocal laser-scanning microscope (Zeiss LSM 710, Carl Zeiss Co., Germany) using an excitation filter of 500 nm and an emission filter of 520 nm. The acquisition settings were maintained throughout the imaging workflow.

### 2-Deoxy-d-Glucose Uptake Assay

Glucose transport was determined by measuring the net uptake of a non-metabolizable analogue of glucose, 2-deoxy-d-glucose (2-DG), as previously described [Rosa et al., 2009]. Briefly, chondrocytes were pre-incubated for 18 h with Glibenclamide (10, 20, 100 nM, and 20 µM) and then incubated for 30 min at 37°C in glucose-free DMEM containing 0.5 mM 2-DG and 0.5 µCi/ml of [2,6-^3^H]-2-DG. Cytochalasin B, a glucose transporter inhibitor (10 µM), was used to determine the non-specific uptake. For each sample, the non-specific uptake was subtracted from the total uptake, before normalization to the respective protein concentration.

### Total RNA Extraction and Quantitative Real-Time RT-PCR (qRT-PCR)

Total RNA was extracted from chondrocyte monolayers using TRIzol® Reagent (Invitrogen, Life Technologies, Co., Paisley, UK) and quantified using a NanoDrop ND-1000 spectrophotometer at 260 nm. Purity and integrity of RNA were assessed as the 240/260 and 280/260 ratios. The cDNA was reverse transcribed using the iScript Select cDNA Synthesis Kit (Bio-Rad), beginning with 1 µg of RNA. Specific sets of primers for GLUT-1, GLUT-3, Kir6.1, Kir6.2, SUR1, SUR2A, SUR2B, and for the housekeeping genes, 18S, GAPDH and L13 (Table I) were designed using Beacon Designer software (PREMIER Biosoft International, Palo Alto, CA). PCR reactions were performed using 25 µg/ml of transcribed cDNA in a final volume of 20 µl.

**TABLE I:** Oligonucleotide Primer Pairs Used for qRT-PCR

Gene name	Genbank accession number	Forward sequence	Reverse sequence
GLUT-1	NM_006516.2	5′-CGT CTT CAT CAT CTT CAC TG -3′	5′-CTC CTC GGG TGT CTT ATC-3′
GLUT-3	NM_006931.2	5′-CGG CTT CCT CAT TAC CTT C-3′	5′-GGC ACG ACT TAG ACA TTG G-3′
Kir6.1	NM_000352	5′-CAA CTG CTG TGT CCA GAT-3′	5′-ATA CGA ATG GTG ATG TTG GA-3′
Kir6.2	NM_ 000525	5′-CAT AGG CAT TAG TGT AGT-3′	5′-TTA TAG AAG AGG CAA CTG-3′
SUR 1	NM_000352	5′-CAA CTG CTG TGT CCA GAT-3′	5′-ATA CGA ATG GTG ATG TTG GA-3′
SUR 2A	NM_005691	5′-AAG CAT TCG GTC ATT GTA G-3′	5′-GCC ACA TAG TAG GTC TGA-3′
SUR 2B	NM_020297	5′-TGG AGA GGA TGT GGA GAA-3′	5′-CTG TAA GAA TGG TGA ATG TGA T-3′
18S rRNA	NM_022551	5′-GAA GAT ATG CTC ATG TGG TGT TG-3′	5′-CTT GTA CTG GCG TGG ATT CTG-3′
RPL13A	NM_012423	5′-GGA AGA GCA ACC AGT TAC TAT GAG-3′	5′-CAG AGT ATA TGA CCA GGT GGA AAG-3′
GAPDH	NM_002046	5′-ACA GTC AGC CGC ATC TTC-3′	5′-GCC CAA TAC GAC CAA ATC C-3′

The efficiency of the amplification reaction for each gene was calculated using a standard curve of a series of diluted cDNA samples, and the specificity of the amplification products was assessed by analyzing the melting curve generated in the process.

Gene expression changes were analyzed using the built-in iQ5 Optical system software v2, which enables the analysis of the results with the Pfaffl method, a variation of the ΔΔC_T_ method corrected for gene-specific efficiencies [Nolan et al., 2006].

The results for each gene of interest were normalized against 18S, the housekeeping gene found to be the most stable under the experimental conditions used, as determined with Genex® software (MultiD Analyses, AB).

### Statistical Analysis

Results are presented as mean ± SEM. Statistical analysis was performed using GraphPad Prism (version 5.00). SPSS software (version 17.0) was used to assess the normality (Kolmogorov–Smirnov test) and homogeneity of variances to determine whether the conditions required to apply parametric tests were satisfied. As in all cases such conditions were observed, the statistical analysis was performed using the paired *t*-test for comparison of each condition with its respective control or one-way ANOVA for multiple comparisons. Data were considered statistically significant at *P* < 0.05.

## RESULTS

### Characterization of the Subunit Composition of K(ATP) Channels Expressed in Human Chondrocytes

**Fig. 1:**
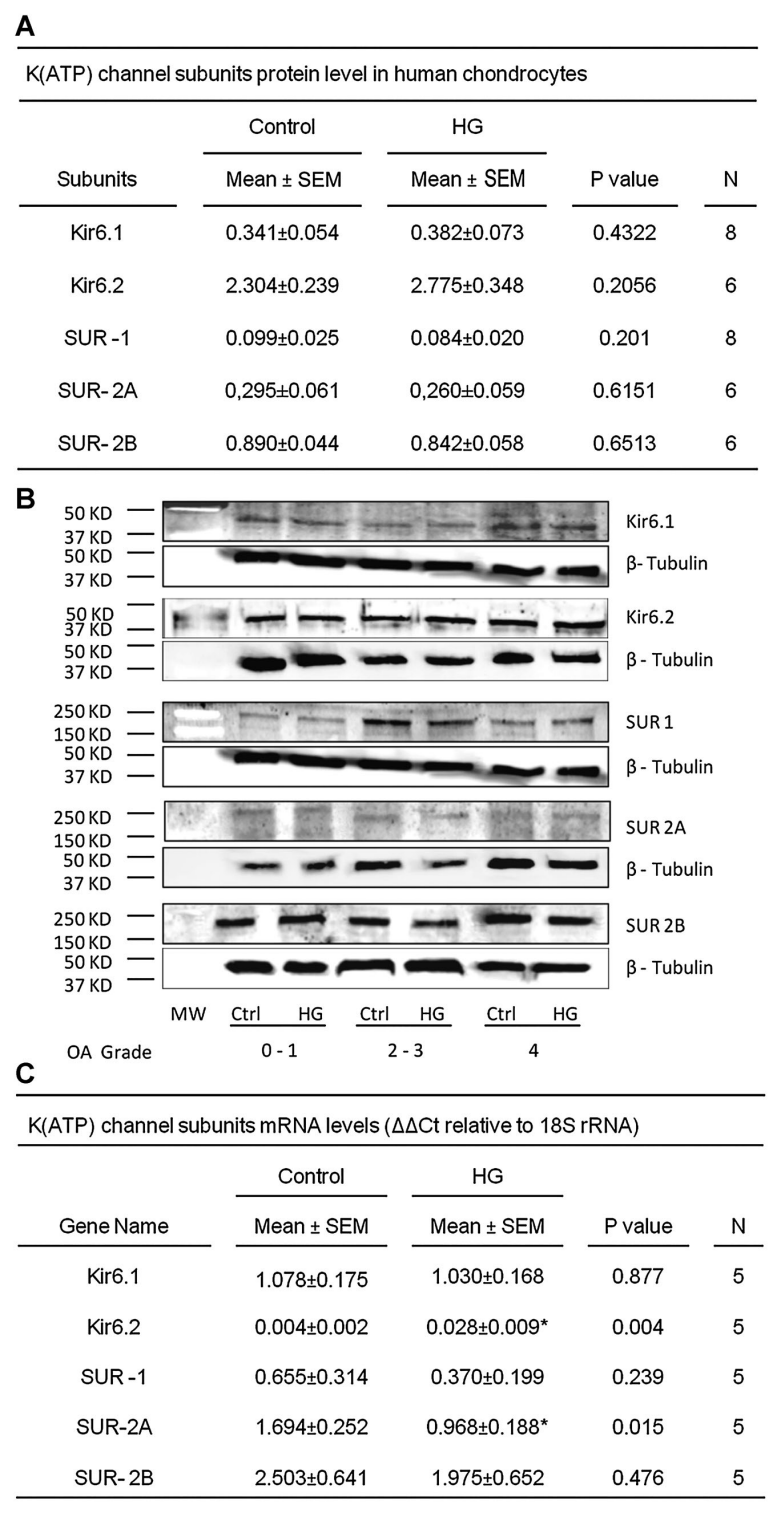
Protein and mRNA levels of K(ATP) channel subunits expressed in human chondrocytes. A: Protein content of each Kir6 and SUR subunit in whole cell extracts from human chondrocytes exposed to high glucose (HG) (30 mM) for 18 h or left untreated (control). The protein content of each subunit was evaluated by Western blotting and normalized to the respective β-tubulin band. B: Western blot images representative of the protein content of each Kir6 and SUR subunit expressed in human chondrocytes according to OA grade, as defined in Materials and Methods Section. MW, molecular weight protein marker. C: mRNA content of each Kir6 and SUR subunit in human chondrocytes exposed to high glucose (HG) (30 mM) for 18 h or left untreated (control). The mRNA content of each subunit was evaluated by qRT-PCR and normalized to the respective 18S rRNA content.

To determine the subunit composition of K(ATP) channels present in human chondrocytes, we evaluated the expression of the different Kir and SUR subunits, both at the protein and mRNA levels, by Western blot and qRT-PCR, respectively. The results obtained show that all the pore-forming and regulatory subunits that can compose K(ATP) channels are expressed in human chondrocytes, although with different intensities and inter-individual variability (Fig. 1). Kir6.1, SUR1, SUR2A, and SUR2B are expressed both at the protein and mRNA levels (Fig. 1). Kir6.2 is apparently the most abundant of the pore-forming subunits at the protein level, but its mRNA is absent or, at least, is expressed at very low levels, which may simply indicate that this protein has a low turnover. On the other hand, even though the mRNAs of all the regulatory SUR subunits were detected in human chondrocytes (Fig. 1C), SUR2B seems to be expressed at the protein level more intensely than SUR2A, while SUR1 was only detected at very low levels (Fig. 1A). Nonetheless, the apparently different intensities of those subunits may simply be accounted for by limitations of the Western blot technique and distinct affinities of the antibodies used. Even though it was not possible to gather a sufficient number of cartilage samples of each OA grade to allow an adequate statistical analysis of the expression of each Kir6 and SUR subunit among the three experimental groups defined, the Western blot images shown in Figure 1B suggest that OA grade does not affect the protein levels of each subunit.

### Immunofluorescence Staining of K(ATP) Channel Subunits in Normal Human Cartilage

To further confirm the results obtained in isolated chondrocytes (Fig. 1), the presence of each subunit in full depth normal (grades 0–1) cartilage samples was assessed by immunofluorescence.

**Fig. 2:**
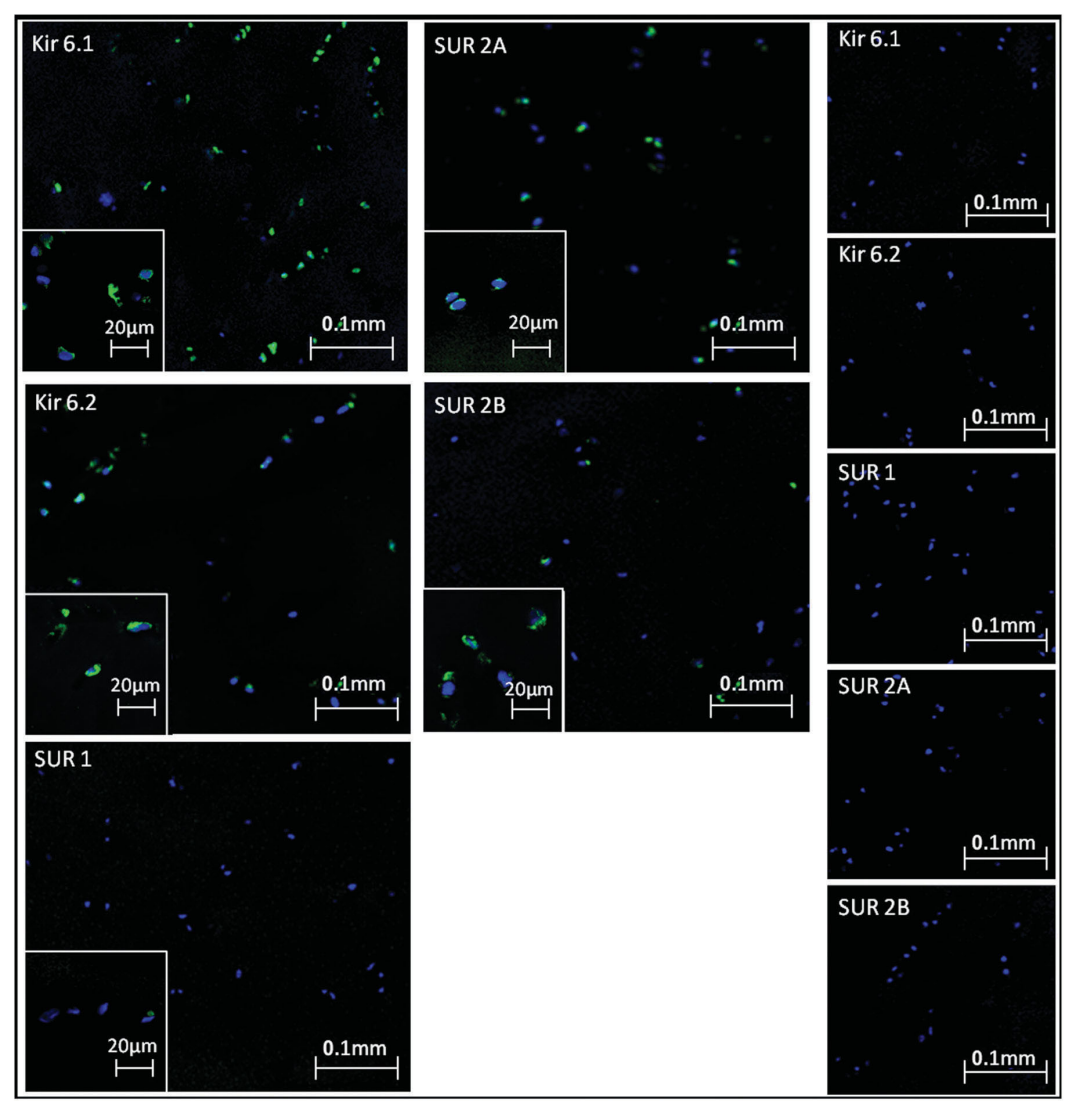
In situ immunofluorescence staining of K(ATP) channel subunits in normal (OA grades 0–1) human cartilage. Each section was stained with an antibody specific for the subunit (green) identified in the upper right corner of the representative image and counterstained with DAPI (blue) to allow visualization of nuclei. Negative controls (column on the right) were obtained by omitting the primary antibody for each subunit. Cartilage sections were viewed and images captured at 100× and 400× (insets) magnifications.

Figure 2 shows the presence of chondrocytes staining positively for Kir6.1, Kir6.2, SUR2A, and SUR2B scattered throughout the cartilage sections and in the midst of non-stained chondrocytes. No cells staining positively for SUR1 could be detected in any of the cartilage sections analyzed. Although no quantitative analysis of the immunofluorescence images was performed, these results are in agreement with those obtained in cell extracts of isolated chondrocytes where SUR1 was detected at very low levels by Western blot, while Kir6.2 and SUR2B seem to be more abundant.

### Role of Exposure to High Glucose on K(ATP) Channel Subunit Protein and mRNA Expression

To elucidate whether exposure to a hyperglycemia-like glucose concentration influences the subunit composition of K(ATP) channels, chondrocytes, classified according to OA grade, were treated with 30 mM glucose for 18 h and the protein and mRNA levels of each subunit were analyzed by Western blot and qRT-PCR, respectively. No differences in MTT reduction capacity were found between chondrocytes of either OA grade, exposed or not to high glucose for 18 h (data not shown), thus showing that exposure to high glucose did not affect chondrocyte viability.

The results presented in Figure 1 show that exposure to high glucose did not significantly affect the protein levels of SUR1, SUR2A, SUR2B, Kir6.1, or Kir6.2 relative to chondrocytes maintained in regular culture medium containing 10 mM glucose. However, as shown in Figure 1C, a small, but statistically significant difference was found in the mRNA level of SUR2A between cells treated with 30 mM glucose (0.97 ± 0.19) and control cells maintained in regular medium (10 mM glucose) (1.69 ± 0.25). On the other hand, the mRNA levels of Kir6.2 were approximately 6.5-fold higher in cells cultured in high glucose for 18 h (0.028 ± 0.009) than in those kept in regular medium (0.004 ± 0.002), although the absolute levels in either condition were very low.

### Regulation of GLUT-1 and GLUT-3 Protein Levels by the K(ATP) Channel Blocker, Glibenclamide

To determine whether the activity of K(ATP) channels is involved in the regulation of glucose transporters in human chondrocytes, the cells were treated for 18 h with glibenclamide, a widely used SUR inhibitor that blocks K(ATP) channels [Burke et al., 2008], in concentrations ranging from 10 nM to 20 µM.

Data presented on Table II show that there are no significant differences in the basal protein levels of GLUT-1 and GLUT3 among the three groups defined according to the macroscopic OA grade, as defined in Materials and Methods Section.

**TABLE II:** Basal Protein Levels of GLUTs-1 and -3 in Human Chondrocytes According to OA Grade

	0–1	2–3	4		
					
OA grade	Mean ± SEM	Mean ± SEM	Mean ± SEM	*P*-value	N
GLUT-1	0.58 ± 0.097	0.70 ± 0.29	0.66 ± 0.21	0.919	6
GLUT-3	1.85 ± 0.58	1.75 ± 0.14	1.49 ± 0.34	0.766	5

**Fig. 3:**
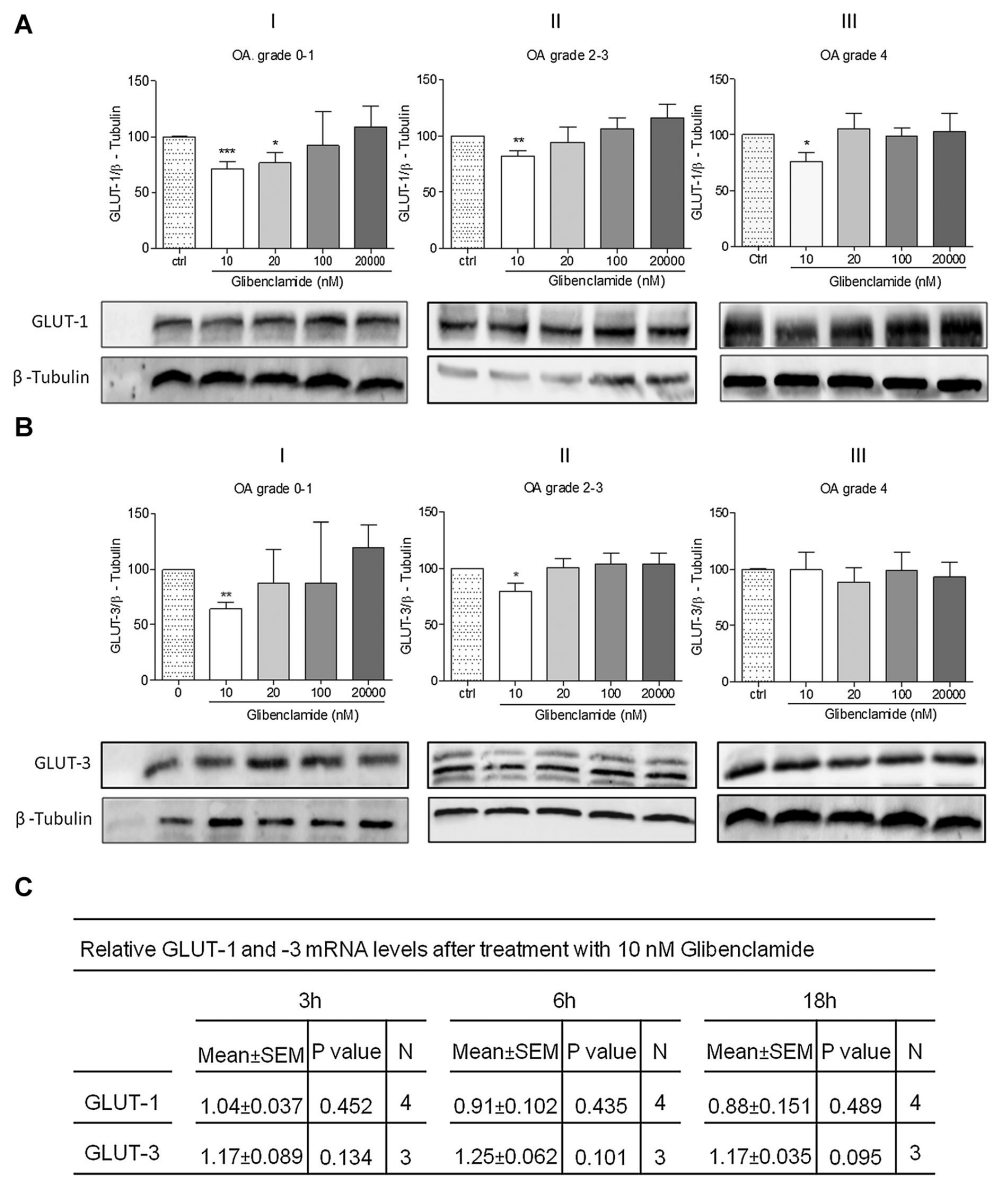
Regulation of total GLUT-1 and GLUT-3 protein content by glibenclamide in chondrocytes from different OA grades. A: Relative GLUT-1 protein levels normalized to the respective β-tubulin band, in whole cell extracts from human chondrocytes of OA grades 0–1 (n = 7), 2–3 (n = 7), and 4 (n = 6) treated with the indicated concentrations of glibenclamide for 18 h. B: Relative GLUT-3 protein levels normalized to the respective β-tubulin band, in whole cell extracts from human chondrocytes of OA grades 0–1 (n = 3), 2–3 (n = 5), and 4 (n = 6) treated with the indicated concentrations of glibenclamide for 18 h. Results are expressed in percentage relative to control untreated cells of the same OA grade ***P < 0.005; **P < 0.01; *P < 0.05 relative to the respective control cells. MW, molecular weight protein marker. C: GLUT-1 and -3 mRNA levels in chondrocytes grades 0–1 treated with 10 nM glibenclamide for the time periods indicated, relative to untreated chondrocytes of the same OA grade.

GLUT-1 protein levels were significantly decreased by treatment of grades 0–1 chondrocytes with glibenclamide 10 nM (71.24 ± 6.59%, *P* = 0.0009, n = 7) and 20 nM (76.55 ± 9.47%, *P* = 0.029, n = 7), whereas only 10 nM was effective in decreasing GLUT-1 protein levels in chondrocytes of OA grades 2–3 (82.16 ± 4.27, *P*= 0.0024, n = 7), and 4 (76.3 ± 8.0, *P* = 0.025, n = 7) (Fig. 3A).

Figure 3B represents the results obtained for GLUT 3. It shows that a significant decrease of GLUT-3 occurs only in chondrocytes from groups with OA grades 0–1 and 2–3 treated with 10 nM glibenclamide (64.17 ± 5.89, *P* = 0.0038; n = 3 and 79.77 ± 7.38, *P* = 0.0265; n = 5, respectively), while no significant differences were found in OA grade 4 chondrocytes with any of the glibenclamide concentrations tested.

Regardless of the OA grade, glibenclamide concentrations above 20 nM had no effect either on GLUT-1 or -3 protein content, although for each OA grade the variability between chondrocytes isolated from different cartilage samples was higher than observed by treatment with 10 nM glibenclamide.

### Role of Glibenclamide in Regulating GLUT-1 and GLUT-3 mRNA Levels in OA Grades 0–1 Chondrocytes

To determine whether the decrease in the protein levels of GLUT-1 and GLUT-3 was due to glibenclamide effects at the transcriptional level, we analyzed the mRNA levels of both glucose transporters by qRT-PCR. Figure 3C shows that there were no statistically significant differences in the mRNA levels of either glucose transporter in glibenclamide-treated groups compared to the control group at any treatment period considered.

### Role of Glibenclamide in Regulating Cellular Glucose Transport Capacity

To determine whether the effects of glibenclamide on GLUT-1 and -3 protein levels had functional consequences in terms of glucose transport capacity by chondrocytes of various OA grades, the uptake of [^3^H]2-DG was measured after treatment with glibenclamide for 18 h. The results obtained (Table III) show no significant differences in 2-DG uptake by cells treated with any of the glibenclamide concentrations tested, compared with the corresponding control untreated chondrocytes of the same OA grade.

**TABLE III:** Relative 2-DG Uptake (% of Untreated Cells)

Glibenclamide (nM)	OA grades 0–1			OA grades 2–3			OA grade 4		
		
	Mean ± SED	*P*-value	N	Mean ± SED	*P*-value	N	Mean ± SED	*P*-value	N
10	92.63 ± 9.79	0.4962	6	106.9 ± 5.98	0.2979	5	94.03 ± 3.14	0.2466	3
20	97.92 ± 1.53	0.2347	6	108.6 ± 8.73	0.3777	4	100.7 ± 4.53	0.8422	3
100	93.95 ± 5.36	0.3322	6	102.2 ± 8.03	0.7749	5	87.33 ± 5.37	0.1629	3
20,000	100.6 ± 3.24	0.8203	6	113.7 ± 8.10	0.1785	4	91.41 ± 8.36	0.4120	3

## DISCUSSION AND CONCLUSIONS

Although the presence of Kir6.1 in human and equine chondrocytes has already been demonstrated [Mobasheri et al., 2007], this study represents the first comprehensive analysis of Kir6.x and SUR subunits expressed in human articular chondrocytes, both at the protein and mRNA levels. The results obtained (Figs. 1 and 2) suggest that Kir6.2 and SUR2B are major K(ATP) channel subunits expressed in human chondrocytes, while Kir6.1 and SUR2A are likely present at much lower levels and SUR1 is barely detectable or even undetectable. How the various Kir and SUR subunits expressed in human chondrocytes assemble to form functional K(ATP) channels is beyond the scope of this study and was not investigated. Nonetheless, it is possible that K(ATP) channels containing more than one SUR subtype co-exist with others containing only one SUR isoform. The relative abundance of each channel type would depend on the expression level of each subunit, as observed in other cells [Chan et al., 2008; Wheeler et al., 2008]. Since Kir 6.2 and SUR2B seem to be more abundant than the other Kir6 and SUR subunits, it seems likely that K(ATP) channels containing these subunits, namely octamers composed of Kir6.2 and SUR2B, predominate in human articular chondrocytes, although all other possible combinations of Kir6 and SUR subunits can also be present. Even though the scarcity of human cartilage samples did not allow us to determine the influence of the OA grade on the subunit composition of K(ATP) channels in human chondrocytes, the results presented in Figure 1 suggest that none of the Kir6 or SUR subunits is affected at the protein level.

The composition of those channels, at least at the protein level, was not significantly affected by culture under a supra-physiologic glucose concentration (30 mM), unlike demonstrated at the mRNA level in other cells, namely mediobasal hypothalamic neurons [Acosta-Martinez and Levine, 2007] and pancreatic β-cells [Moritz et al., 2001].

The nucleotide binding affinity of SUR2B is much higher than that of SUR2A, but lower than that of SUR1, although its channel gating activity is similar to that of SUR1 [Matsuo et al., 2000]. Therefore, SUR2B-containing K(ATP) channels are functionally very similar to those that contain SUR1, namely those expressed in pancreatic β-cells [Burke et al., 2008] and in mediobasal hypothalamic neurons [Acosta-Martinez and Levine, 2007] that readily bind ATP in response to augmented glucose availability. To test the hypothesis that K(ATP) channels expressed in human chondrocytes behave similarly, we attempted to measure changes in membrane potential induced by culture under a supra-physiologic glucose concentration (30 mM) which is in the range of plasma glucose concentrations found in diabetic patients. For this, we loaded the cells with the potential-sensitive fluorescence dye, bis(1,3-dibutylbarbituric acid)trimethine oxonol [DiBAC 4 (3)] that becomes fluorescent upon depolarization. Unfortunately, we were unable to detect any changes using fluorescence spectroscopy, probably because changes in membrane potential occur in a very dynamic and fast manner. Possibly, in vivo fluorescence imaging may be required to detect such fast and transient changes. Therefore, we were unable to directly demonstrate whether glucose availability affects the chondrocyte membrane potential.

To further test the hypothesis that K(ATP) channels function as metabolic sensors in human chondrocytes playing a role in the modulation of glucose transport capacity, we used glibenclamide, a widely used SUR inhibitor, to block these channels and measured the whole cell content of major glucose transporters, GLUT-1 and GLUT-3. Glibenclamide displays differential selectivity towards distinct SUR subunits, being moderately selective for SUR1 [Burke et al., 2008]. The sensitivity of equine chondrocytes to glibenclamide is lower than that found in cells expressing SUR1 and in the same range of that presented by cells in which K(ATP) channels contain SUR2B [Mobasheri et al., 2007] which further suggests that K(ATP) channels containing this subunit are relevant in human chondrocytes. Nevertheless, as K(ATP) channels composed of other subunits may also exist in human chondrocytes, we used glibenclamide concentrations ranging from 10 nM to 20 µM in order to cover the whole range of channel sensitivities to this SUR inhibitor. Interestingly, only the lowest concentration was effective in reducing GLUT-1 protein content in chondrocytes from all OA grades, whereas 20 nM was effective only in grades 0–1 OA chondrocytes (Fig. 3A). GLUT-3 content, however, was only reduced by treatment of grades 0–1 and 2–3, but not grade 4 OA chondrocytes with glibenclamide 10 nM (Fig. 3B). These results indicate that closing K(ATP) channels, at least partially, is sufficient to reduce the cell protein content of two major glucose transporters, GLUT-1 and GLUT-3, without affecting their mRNA levels, in normal human chondrocytes, in a way similar to the reduction induced by exposure to 30 mM glucose [Rosa et al., 2009].

The inability of OA chondrocytes to downregulate the protein levels of GLUT-1 and GLUT-3 in response to glibenclamide (Fig. 3), mimicking their inability to adjust to high extracellular glucose concentrations [Rosa et al., 2009], further indicates that either K(ATP) channels are functionally defective or some other downstream component or process is impaired in OA chondrocytes. Nonetheless, the possibility that ATP production may simultaneously be impaired in advanced OA chondrocytes cannot be excluded.

Another intriguing question arises from the observation that the response to glibenclamide in terms of GLUT-1 and GLUT-3 protein content, occurred only with the lowest glibenclamide concentrations tested. In equine chondrocytes, similar glibenclamide concentrations (10–20 nM) elicited a partial reduction in current amplitude, while much higher concentrations in the range of 100–300 nM were required to completely block K(ATP) channel activity [Mobasheri et al., 2007]. Therefore, it is possible that different ranges of channel activity elicit distinct changes in membrane potential that operate diverse signaling pathways and cell responses. This is further supported by the observation that K(ATP) channels with distinct subunit compositions and therefore with different glibenclamide and nucleotide sensitivities, may coexist in human chondrocytes (Figs. 1 and 2).

Nonetheless, glibenclamide-induced changes in the total protein content of GLUT-1 and GLUT-3 were not accompanied by corresponding decreases in glucose transport capacity by chondrocytes of any OA grade (Table III). This suggests that even though K(ATP) channel activity regulates the availability of GLUT-1 and GLUT-3, other mechanisms are involved in the regulation of the overall glucose transport capacity of human chondrocytes. On one hand, other glucose transporters known to be expressed in chondrocytes [Mobasheri et al., 2008] may not be regulated by K(ATP) channels and compensate for changes in GLUT-1 and -3. On the other hand, GLUT-1 has been shown to exist in a dynamic equilibrium involving the plasma membrane, intracellular storage in the trans Golgi network and lysosomal degradation [Ortiz et al., 1992]. Our previous study indicated that high glucose-induced GLUT-1 downregulation involved lysosomal degradation [Rosa et al., 2009]. Thus, it is possible that K(ATP) channel activity affects total GLUT-1 and possibly GLUT-3 protein content by increasing lysosomal degradation of intracellular stores, in agreement with studies in other cells suggesting that GLUT-1 degradation and functional membrane expression may be regulated through independent pathways [Ortiz et al., 1992].

In summary, this study contributes to elucidate the subunit composition of K(ATP) channels expressed in human chondrocytes and establishes a role for these channels as components of the glucose sensing apparatus in these cells by demonstrating that their activity influences the abundance of two major glucose transporters, GLUT-1 and GLUT-3. Nonetheless, since glucose transport was not similarly affected, additional mechanisms must be involved in the adaptation of human chondrocytes to high extracellular glucose concentrations. Moreover, this study also shows that chondrocytes isolated from cartilage of increasing OA grade are less responsive to changes in K(ATP) channel activity, which may contribute to the inability of OA chondrocytes to downregulate glucose transporters and avoid the deleterious effects of high extracellular glucose concentrations that we have previously observed [Rosa et al., 2009, 2011]. Taken together, the results presented highlight the importance of K(ATP) channels as metabolic sensors and potential components of a broad glucose sensing apparatus that allows human chondrocytes to adjust to varying extracellular glucose concentrations.

## Abbreviations

2-DG: 2-deoxy-d-glucose
DiBAC 4(3): bis(1,3-dibutylbarbituricacid)trimethine oxonol
GLUT: glucose transporter
K(ATP): channel, ATP-sensitive potassium channel
OA: osteoarthritis
qRT-PCR: real-time RT-PCR
SUR: sulfonylurea receptor
